# Which Learning Domains Benefit Most from Occupational Safety-Integrated Project-Based Learning? A Comparative Analysis of Cognitive, Affective, and Psychomotor Outcomes in Agricultural Vocational Education

**DOI:** 10.12688/f1000research.187948.1

**Published:** 2026-08-06

**Authors:** Basma Basma, Patang Patang, Suardi Suardi, Muhammad Irfan, Darmawan Darmawan, Ahmad Swandi

**Affiliations:** 1 Doctoral Program in Educational Sciences, Postgraduate Program, State University of Makassar, Makassar, South Sulawesi, Indonesia; 2 Department of Agricultural Technology, State University of Makassar, Makassar, South Sulawesi, Indonesia; 3 Non-formal Education Study Program, State University of Makassar, Makassar, South Sulawesi, Indonesia; 4 Primary Teacher Education Study Program, State University of Makassar, Makassar, South Sulawesi, Indonesia; 5 Plantation Crop Production Technology Study Program, Politeknik Pertanian Negeri Pangkajene Kepulauan, Pangkep, Sulawesi, Indonesia; 6 Science Education Study Program, Bosowa University, Makassar, South Sulawesi, Indonesia

**Keywords:** Occupational Safety, Project-Based Learning, Vocational Education, Holistic Learning Outcomes, Agricultural Education, Authentic Learning.

## Abstract

Occupational safety has become an essential competency in vocational education, particularly in agricultural programs where students are exposed to high-risk practical activities. Although Project-Based Learning (PjBL) has been widely recognized for promoting authentic learning, limited evidence exists regarding how Occupational Safety-Integrated Project-Based Learning (OS-PjBL) influences different learning domains simultaneously. This study investigated the comparative learning outcomes of OS-PjBL across the cognitive, affective, and psychomotor domains in agricultural vocational education. A quantitative evaluative design was employed involving 30 undergraduate students enrolled in the Agricultural Production Technology program at Pangkep State Polytechnic of Agriculture, Indonesia. The validated OS-PjBL model was implemented through authentic project activities integrating hazard identification, risk assessment, safety procedures, and reflective practice. Cognitive outcomes were measured using achievement tests, while affective and psychomotor competencies were assessed through structured observation sheets and performance rubrics. Data were analyzed descriptively using measures of central tendency and percentage-based comparisons across learning domains. The findings revealed substantial improvements across all three domains. Students achieved a post-test cognitive mean score of 86.7, while affective and psychomotor competencies reached overall achievement levels of 93% and 95%, respectively. Comparative analysis indicated that psychomotor competence demonstrated the highest level of achievement, followed by affective and cognitive outcomes. These findings suggest that integrating occupational safety throughout project-based learning effectively promotes holistic competence development by simultaneously strengthening conceptual understanding, professional attitudes, and practical workplace performance. This study extends the PjBL literature by positioning occupational safety not merely as supplementary instructional content but as the pedagogical organizing principle of authentic learning experiences, providing a practical framework for preparing safety-conscious and industry-ready vocational graduates.

## 1. Introduction

Vocational education is designed to prepare graduates with practical competencies that meet the evolving demands of industry and the workplace ( Tarkan Düzgünçınar, 2025). Unlike general higher education, vocational programs emphasize authentic learning experiences through laboratory practice, workshops, fieldwork, and industry-based projects that closely resemble professional environments ( De Bruijn & Leeman, 2011; Rintala & Nokelainen, 2020). These practical learning experiences enable students to acquire technical expertise while developing the professional competencies required for successful employment ( Simon, 2024). However, the increasing emphasis on hands-on learning also exposes students to occupational hazards that require systematic safety education throughout the learning process.

Among various vocational disciplines, agricultural education represents one of the most hazardous learning environments. Students are routinely involved in activities requiring the operation of agricultural machinery, the handling of chemical substances, field experimentation, and outdoor practical work under diverse environmental conditions. Such activities expose students to multiple risks, including mechanical injuries, chemical exposure, ergonomic hazards, and environmental accidents ( Alghamdi et al., 2026; Das et al., 2025). Consequently, occupational safety should no longer be viewed merely as a regulatory requirement but rather as a fundamental professional competence that vocational graduates must demonstrate before entering the workforce ( Zabolotnii & Fedorina, 2026).

Recent international discussions on Technical and Vocational Education and Training (TVET) have increasingly emphasized that workplace readiness extends beyond technical proficiency ( Kamaruzaman et al., 2025). Modern industries expect graduates to possess safety awareness, hazard identification skills, risk assessment abilities, and responsible workplace behavior ( Davis et al., 2021). In agricultural industries, where occupational accidents remain relatively high, developing these competencies during vocational education becomes particularly important ( Lawver et al., 2024). Therefore, educational institutions are expected to integrate occupational safety not as an isolated subject but as an essential component of authentic learning experiences that cultivate professional judgment and safe working practices.

Despite this expectation, many vocational institutions continue to implement occupational safety instruction primarily through classroom-based theoretical approaches. As reported in preliminary observations at Pangkep State Polytechnic of Agriculture, students generally understand occupational safety as a set of rules rather than as a professional culture guiding every practical activity. This situation creates a gap between industry expectations and students’ actual competencies, indicating the need for instructional approaches capable of simultaneously strengthening knowledge, attitudes, and practical safety skills.

Project-Based Learning (PjBL) has become one of the most widely adopted instructional approaches in vocational education because it places students in authentic situations that require them to solve complex real-world problems collaboratively ( Martawijaya, Rahmadhanningsih, et al., 2023a; Martawijaya, Swandi, et al., 2023; Tan et al., 2025). Rather than passively receiving information, students actively construct knowledge while designing solutions, managing projects, communicating with peers, and reflecting upon their learning experiences ( Ho, 2024; Koohang et al., 2016). These characteristics make PjBL particularly suitable for vocational education, where professional competence develops through meaningful engagement with practical tasks.

The pedagogical foundation of PjBL aligns closely with experiential learning and Dewey’s principle of *learning by doing*, emphasizing that meaningful learning occurs through direct engagement with authentic activities ( Kolb & Kolb, 2022; Lavado-Anguera et al., 2024). By working on realistic projects, students connect theoretical concepts with practical applications, enabling deeper conceptual understanding while simultaneously strengthening higher-order thinking, collaboration, communication, and problem-solving skills ( Mebert et al., 2020). These characteristics explain why PjBL has been increasingly recognized as an effective approach for preparing graduates capable of responding to dynamic workplace challenges.

Within occupational safety education, PjBL offers an additional advantage because safety procedures become embedded within authentic project activities rather than being taught as isolated theoretical content ( Dwi Astartia et al., 2024). Students are required to identify hazards, assess potential risks, apply standard operating procedures, and evaluate safety practices throughout project implementation ( Wasilkiewicz Edwin et al., 2024). Consequently, occupational safety becomes part of students’ everyday decision-making process instead of remaining a collection of memorized regulations. Such authentic integration has the potential to transform safety education from compliance-oriented instruction into competence-oriented learning.

The effectiveness of vocational education should not be evaluated solely through students’ acquisition of conceptual knowledge. According to Bloom’s taxonomy and subsequent educational frameworks, meaningful learning encompasses three interconnected domains: cognitive, affective, and psychomotor development ( Dettmer, 2005). These domains collectively represent holistic competence, which is particularly essential in vocational education where graduates are expected to demonstrate knowledge, professional attitudes, and practical performance simultaneously.

The cognitive domain reflects students’ conceptual understanding and problem-solving abilities related to occupational safety principles ( Liang et al., 2022). In agricultural vocational education, this includes understanding hazard identification, risk assessment, safe equipment operation, and emergency procedures. However, possessing knowledge alone does not necessarily guarantee safe workplace behavior.

The affective domain represents students’ values, attitudes, responsibility, and commitment toward occupational safety. Developing safety awareness is particularly important because students who value workplace safety are more likely to comply with safety regulations, demonstrate responsible behavior, and contribute to the development of a positive safety culture within professional environments ( Darmawang et al., 2024; Guerin & Toland, 2020).

Meanwhile, the psychomotor domain concerns students’ ability to translate knowledge and attitudes into safe practical performance. Competence in using personal protective equipment, operating machinery safely, conducting risk inspections, and implementing standard operating procedures represents the behavioral manifestation of occupational safety competence. Since vocational graduates are ultimately evaluated through workplace performance, psychomotor competence remains a critical indicator of instructional success.

These three domains should not be viewed as independent outcomes but as complementary dimensions of professional competence. Effective occupational safety education therefore requires instructional approaches capable of simultaneously promoting conceptual understanding, strengthening safety attitudes, and improving practical workplace performance.

Although Project-Based Learning has consistently been reported to improve students’ learning outcomes across various educational contexts, previous studies have predominantly evaluated its effectiveness using overall academic achievement or general competency scores. Much less attention has been devoted to understanding how different learning domains respond to Project-Based Learning interventions, particularly when occupational safety constitutes the primary learning context.

This limitation is particularly evident within agricultural vocational education, where occupational safety competence is inherently multidimensional. Students are expected not only to understand safety concepts but also to internalize safety values and consistently demonstrate safe workplace practices. Nevertheless, existing studies rarely investigate whether Project-Based Learning benefits cognitive, affective, and psychomotor outcomes equally or whether certain domains experience greater improvement than others. Consequently, evidence explaining the differential impact of Project-Based Learning across learning domains remains limited.

Understanding these differences is important because instructional strategies may influence knowledge acquisition, attitude formation, and practical skill development through different learning mechanisms. Identifying which learning domain benefits most from Occupational Safety-Integrated Project-Based Learning can provide stronger theoretical explanations regarding how authentic project-based experiences contribute to holistic competence development in vocational education.

Against this background, the present study investigates the comparative effects of Occupational Safety-Integrated Project-Based Learning on students’ cognitive, affective, and psychomotor learning outcomes in agricultural vocational education. Rather than merely examining whether Project-Based Learning improves learning outcomes, this study seeks to identify which learning domain benefits most from the instructional intervention. By comparing improvements across the three domains, the study contributes empirical evidence toward understanding the multidimensional nature of occupational safety competence and offers practical implications for designing more effective vocational learning environments. Accordingly, this study addresses the following research questions:

RQ1. How does Occupational Safety-Integrated Project-Based Learning influence students’ cognitive, affective, and psychomotor learning outcomes?

RQ2. Which learning domain demonstrates the highest level of achievement following the implementation of Occupational Safety-Integrated Project-Based Learning?

## 2. Literature review

### 2.1 Occupational safety as a core competency in vocational education

Vocational education is fundamentally designed to prepare graduates who are capable of performing professional tasks safely, efficiently, and responsibly within authentic workplace environments. Consequently, occupational safety is increasingly recognized not merely as a regulatory obligation but as a core professional competency that should be systematically developed throughout vocational education. Modern industries expect graduates to possess not only technical expertise but also the ability to recognize workplace hazards, assess operational risks, comply with safety procedures, and make informed decisions that minimize accidents while maintaining productivity. This expectation reflects a broader shift in vocational education from emphasizing task completion toward developing comprehensive workplace competence that integrates technical performance with occupational safety.

Occupational safety competence is inherently multidimensional because it combines cognitive understanding, professional attitudes, and practical performance within authentic work situations. From a cognitive perspective, learners must understand workplace hazards, risk assessment procedures, emergency responses, and relevant occupational safety regulations ( KV et al., 2023). However, knowledge alone is insufficient to ensure safe workplace behaviour. Students must also develop positive attitudes toward safety by demonstrating responsibility, discipline, compliance with safety procedures, and awareness of the potential consequences of unsafe practices. These affective attributes subsequently influence the psychomotor dimension, where learners are expected to apply safety principles consistently during practical activities through the correct use of personal protective equipment (PPE), implementation of standard operating procedures (SOPs), hazard control, and safe equipment operation ( Xu et al., 2018). Accordingly, occupational safety competence should be viewed as an integrated construct in which knowledge, attitudes, and behaviour interact continuously during workplace practice.

Within agricultural vocational education, the importance of occupational safety becomes even more pronounced due to the complexity of workplace hazards encountered during practical learning. Students routinely operate agricultural machinery, handle chemical substances such as pesticides and fertilizers, perform fieldwork under varying environmental conditions, and interact with biological agents that may threaten health and safety. These authentic learning environments provide valuable opportunities for competency development but simultaneously expose students to multiple occupational risks. Therefore, agricultural vocational institutions are required to implement instructional approaches that prepare students not only to perform technical tasks but also to anticipate hazards, evaluate risks, and consistently apply safe working practices throughout project implementation ( Morris et al., 2024).

Previous studies have consistently reported that occupational safety education contributes positively to students’ workplace readiness ( Qin & MANLY, 2024), safety awareness ( Şenkal et al., 2021), and accident prevention ( Boini et al., 2017). Learners who receive systematic safety instruction generally demonstrate stronger hazard identification skills, greater compliance with safety procedures, and more responsible workplace behaviour than those receiving limited safety training. Nevertheless, much of the existing literature continues to conceptualize occupational safety education as the delivery of safety regulations or procedural knowledge through lectures, demonstrations, or isolated training sessions ( Bęś & Strzałkowski, 2024). While these approaches successfully improve conceptual understanding, they often provide limited opportunities for learners to internalize safety principles through repeated authentic practice. Consequently, the development of occupational safety competence frequently remains fragmented, with cognitive achievement receiving greater emphasis than affective development and practical performance.

These limitations indicate the need for instructional approaches capable of embedding occupational safety within meaningful learning experiences rather than treating safety as a separate instructional topic. Such approaches should enable students to learn safety principles while simultaneously solving authentic workplace problems, collaborating with peers, making professional decisions, and reflecting upon their experiences. Project-Based Learning offers considerable potential for achieving these objectives because it situates occupational safety within realistic vocational tasks where knowledge, professional attitudes, and practical competencies develop concurrently. This perspective provides the conceptual foundation for examining Occupational Safety-Integrated Project-Based Learning as an instructional strategy for promoting holistic competence development in vocational education.

### 2.2 Project-based learning and authentic learning theory

Project-Based Learning (PjBL) has become one of the most influential instructional approaches in vocational education because it emphasizes authentic problem solving through sustained student engagement in meaningful projects ( Sudira et al., 2022; Tan et al., 2025). Unlike conventional teacher-centred instruction, PjBL positions learners as active participants responsible for investigating problems, planning solutions, implementing projects, evaluating outcomes, and reflecting upon their learning experiences ( Martawijaya, Rahmadhanningsih, et al., 2023a). Through these activities, students construct knowledge collaboratively while applying theoretical concepts to situations that closely resemble professional practice. Consequently, learning becomes a process of active knowledge construction rather than passive information acquisition.

The pedagogical foundations of Project-Based Learning are strongly associated with experiential learning theory, which proposes that meaningful learning occurs through the transformation of experience into knowledge ( Kokotsaki et al., 2016). According to experiential learning, students learn most effectively when they actively engage in concrete experiences, reflect upon those experiences, develop conceptual understanding, and subsequently apply newly acquired knowledge to different situations ( Glaze, 1999). Within Project-Based Learning, this cycle is reflected through authentic project implementation, collaborative discussion, evaluation of project outcomes, and continuous refinement of professional practice. As a result, students are encouraged not only to understand theoretical concepts but also to develop procedural knowledge that can be transferred to real workplace situations.

Project-Based Learning is also closely aligned with Dewey’s principle of *learning by doing*, which emphasizes that education should be grounded in meaningful activities connected to real-life experiences ( Melkieus J, 2026). Dewey argued that knowledge develops through purposeful action rather than through passive memorization of information. This principle is particularly relevant to vocational education, where professional competence depends upon learners’ ability to integrate theoretical understanding with practical performance. By requiring students to solve authentic workplace problems, Project-Based Learning enables them to develop technical expertise while simultaneously strengthening communication, collaboration, decision-making, and professional responsibility.

Another important theoretical perspective supporting Project-Based Learning is situated learning, which argues that knowledge is constructed within the social and contextual environments in which it is applied ( Chang et al., 2024). Learning therefore becomes more meaningful when students participate in authentic professional practices that resemble the situations they are likely to encounter after graduation. Within vocational education, authentic projects provide opportunities for learners to experience workplace complexity while interacting with peers, instructors, and industry-relevant problems. Such experiences facilitate the development of professional judgement because students learn not only what to do but also why particular decisions are appropriate within specific workplace contexts.

Numerous empirical studies have demonstrated the educational benefits of Project-Based Learning across diverse educational settings. Previous research consistently reports improvements in conceptual understanding, higher-order thinking skills, collaboration, motivation, communication, and practical competence following participation in authentic project activities. Meta-analytic evidence similarly indicates that Project-Based Learning is particularly effective in vocational and STEM education because it provides repeated opportunities for learners to connect theoretical concepts with practical application ( Ibrahim, 2025; Martawijaya, Rahmadhanningsih, et al., 2023b; Swandi et al., 2022). However, most existing studies evaluate Project-Based Learning using overall academic achievement or general competency measures, providing relatively limited evidence regarding how individual learning domains respond differently to project-based experiences.

Within occupational safety education, the application of Project-Based Learning remains comparatively underexplored. Existing instructional approaches frequently introduce occupational safety as supplementary content delivered before or during practical activities, rather than integrating safety principles throughout the entire learning process. Consequently, learners may perceive occupational safety as a procedural requirement instead of an essential component of professional decision-making. Embedding occupational safety into every phase of project implementation—including hazard identification, risk assessment, project planning, implementation, monitoring, evaluation, and reflection—has the potential to transform safety education into an authentic learning experience that simultaneously strengthens conceptual understanding, professional attitudes, and practical competence. This integrated perspective provides the theoretical rationale for the Occupational Safety-Integrated Project-Based Learning model examined in the present study.

### 2.3 Holistic learning outcomes in vocational education

The effectiveness of vocational education should be evaluated through a holistic perspective that extends beyond students’ academic achievement. Unlike general education, vocational education aims to prepare graduates who are capable of applying knowledge, demonstrating professional attitudes, and performing practical tasks in authentic workplace environments. Consequently, learning outcomes should reflect the integrated development of cognitive, affective, and psychomotor competencies, which together represent professional competence. This perspective aligns with contemporary competency-based education, emphasizing that successful vocational graduates must possess not only conceptual understanding but also the values and practical abilities required for safe and effective workplace performance ( Janssens et al., 2023).

The cognitive domain represents students’ conceptual understanding of occupational safety principles and their ability to apply knowledge in solving workplace-related problems ( Evains et al., 2024; Liang et al., 2022). Within agricultural vocational education, cognitive competence includes understanding hazard identification, risk assessment, emergency procedures, safe machinery operation, and occupational safety regulations. Strong cognitive achievement enables students to recognize workplace hazards and make informed decisions before engaging in practical activities. Nevertheless, conceptual understanding alone cannot guarantee safe workplace behaviour because learners may possess adequate knowledge without consistently applying it during authentic work situations.

The affective domain reflects students’ attitudes, values, motivation, and professional commitment toward occupational safety. In vocational education, affective learning is particularly important because safety-related behaviours are strongly influenced by students’ willingness to comply with procedures, assume responsibility, collaborate with colleagues, and maintain awareness of workplace risks ( Madsgaard et al., 2022). The development of positive safety attitudes contributes directly to the establishment of a safety culture, in which occupational safety becomes an internalized professional value rather than an externally imposed requirement ( Edgar et al., 2021). Consequently, affective learning plays a central role in ensuring that safety knowledge is translated into responsible workplace behaviour.

The psychomotor domain concerns learners’ ability to perform occupational safety procedures accurately within authentic working environments ( Saleem et al., 2022). Competence in the correct use of personal protective equipment (PPE), hazard control, implementation of standard operating procedures (SOPs), and safe equipment operation represents the practical manifestation of occupational safety competence ( Radu et al., 2026). Since vocational graduates are ultimately evaluated through their workplace performance, psychomotor competence serves as a critical indicator of instructional effectiveness. Authentic project experiences therefore provide important opportunities for students to repeatedly practice occupational safety procedures until safe behaviour becomes part of their professional routine.

Although these three domains represent different dimensions of learning, they should not be interpreted as independent competencies. Cognitive understanding provides the foundation for informed decision-making, affective development shapes learners’ commitment toward safe practice, while psychomotor competence demonstrates the successful application of knowledge and attitudes in authentic workplace situations. The interaction among these domains reflects the holistic nature of vocational competence, suggesting that effective instructional approaches should facilitate balanced development across all three dimensions rather than emphasizing academic achievement alone.

Previous research has generally reported positive effects of Project-Based Learning on students’ learning outcomes; however, most studies evaluate instructional effectiveness using overall achievement scores or general competency indicators. Relatively few investigations examine how cognitive, affective, and psychomotor outcomes develop simultaneously following project-based instruction, particularly within occupational safety education. Consequently, evidence explaining whether authentic project experiences influence these three learning domains equally remains limited. Understanding these differences is particularly important because vocational learning requires instructional approaches capable of balancing conceptual understanding, professional attitudes, and workplace performance within authentic educational settings.

### 2.4 Research Gap and Conceptual Framework

Research conducted over the past two decades consistently demonstrates that Project-Based Learning contributes positively to students’ academic achievement, collaborative skills, problem-solving ability, and practical competence across various educational contexts. Similarly, occupational safety education has been widely recognized as an essential component of vocational training because it strengthens workplace readiness, hazard awareness, and professional responsibility. Collectively, these studies suggest that both Project-Based Learning and occupational safety instruction contribute significantly to the preparation of competent vocational graduates.

Despite these advances, based on Table 1, several important gaps remain within the existing literature. First, most Project-Based Learning studies evaluate instructional effectiveness using overall learning achievement without distinguishing how different learning domains respond to authentic project experiences. Second, occupational safety education continues to be implemented predominantly as separate instructional content rather than being integrated throughout the entire learning process. Consequently, relatively little empirical evidence explains whether embedding occupational safety within Project-Based Learning promotes balanced development of cognitive, affective, and psychomotor competencies or whether particular domains benefit more substantially than others.

**Table 1.** Summary of previous studies on project-based learning and occupational safety.

Authors	Context	Main findings	Remaining gap
Chiu (2024)	General education	PjBL improves authentic learning	Did not examine occupational safety
Bell (2010)	STEM education	Improved collaboration and problem solving	Did not compare learning domains
Ospankulova et al. (2025)	Higher education	PjBL enhances student engagement	No vocational safety context
TVET studies	Vocational education	Improved practical competence	Focused mainly on technical skills
Occupational safety education studies	Safety training	Increased safety awareness	Predominantly lecture-based; limited integration with PjBL
Present study	Agricultural vocational education	Comparison of cognitive, affective, and psychomotor outcomes within OS-PjBL	Addresses the identified gaps

These limitations are particularly evident within agricultural vocational education, where occupational safety competence constitutes a fundamental professional requirement due to the high-risk nature of agricultural work. Students are expected not only to understand occupational safety principles but also to demonstrate responsible workplace behaviour and consistently implement safe working procedures during practical activities. However, empirical studies simultaneously examining these three dimensions within agricultural vocational contexts remain scarce. As a result, current understanding of how Occupational Safety-Integrated Project-Based Learning supports holistic competence development remains incomplete.

The present study addresses these gaps by investigating the comparative learning outcomes of Occupational Safety-Integrated Project-Based Learning across the cognitive, affective, and psychomotor domains. Rather than examining overall instructional effectiveness alone, this study seeks to identify which learning domain benefits most from authentic occupational safety project experiences. By focusing on agricultural vocational education, the study also extends previous Project-Based Learning research into a context characterized by complex workplace hazards and strong demands for safety competence. Accordingly, this study contributes to the literature in three important ways. First, it conceptualizes occupational safety as an organizing principle of project-based learning rather than as supplementary instructional content. Second, it provides a comparative analysis of cognitive, affective, and psychomotor learning outcomes following the implementation of Occupational Safety-Integrated Project-Based Learning. Third, it offers empirical evidence from agricultural vocational education, a context that remains underrepresented within contemporary Project-Based Learning research.

**Figure 1.**
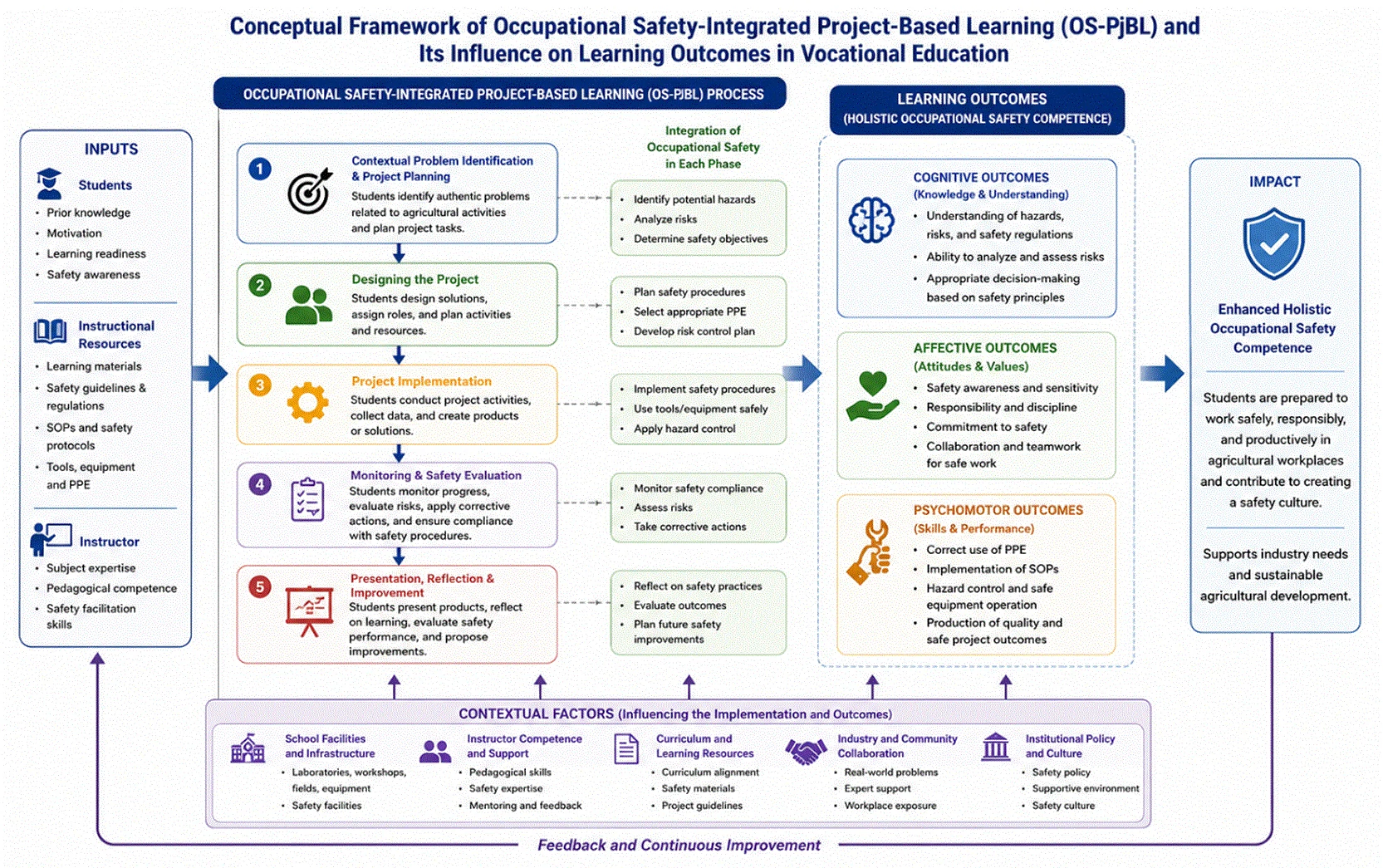
Conceptual framework.

Based on the theoretical perspectives and empirical findings discussed above, Figure 1 presents the conceptual framework guiding the present study. The framework proposes that Occupational Safety-Integrated Project-Based Learning creates authentic learning experiences through hazard identification, collaborative problem solving, implementation of safety procedures, and reflective practice. These learning processes are expected to promote holistic occupational safety competence by simultaneously strengthening students’ cognitive understanding, professional attitudes, and psychomotor performance within authentic vocational learning environments.

## 3. Method

### 3.1 Research design

This study employed a quantitative evaluative approach to examine the learning outcomes resulting from the implementation of an Occupational Safety-Integrated Project-Based Learning (OS-PjBL) model in agricultural vocational education. This study employed one-group implementation design using descriptive comparative analysis to examine cognitive, affective, and psychomotor learning outcomes following the implementation of Occupational Safety-Integrated Project-Based Learning. The study was derived from a broader Educational Design Research project that adopted the ADDIE (Analysis, Design, Development, Implementation, and Evaluation) model to develop and validate the instructional model. However, the present article does not report the entire development process. Instead, it focuses exclusively on the implementation phase by comparing students’ cognitive, affective, and psychomotor learning outcomes following the application of the validated learning model.

The implementation stage was selected because it provides empirical evidence regarding how different learning domains respond to Occupational Safety-Integrated Project-Based Learning. Rather than evaluating the validity or practicality of the instructional model, this study emphasizes the comparative improvement of learning outcomes across the three domains of learning, thereby providing a more comprehensive understanding of holistic competence development in vocational education.

### 3.2 Participants

This research was conducted from September 2025 to December 2025. The participants in this study were 30 undergraduate students (aged 19–21 years) from the Department of Agricultural Production Technology, Pangkep State Polytechnic of Agriculture, Indonesia, who participated in the field implementation phase of the Occupational Safety-Integrated Project-Based Learning (OS-PjBL) model. The participants represented one intact class enrolled in practical agricultural courses where occupational safety and health (OSH) competencies constitute essential learning outcomes.

Participants were selected using an intact-class purposive sampling approach because they were actively engaged in laboratory activities, field practice, and agricultural equipment operation, all of which require the application of occupational safety procedures. The field implementation involved an entire class of 30 students to provide stronger empirical evidence regarding the effectiveness of the Occupational Safety-Integrated Project-Based Learning (OS-PjBL) model and students’ cognitive, affective, and psychomotor learning outcomes under authentic vocational learning conditions.

Throughout the intervention, students completed a sequence of authentic project-based learning activities integrating occupational safety principles, including hazard identification, risk assessment, development of standard operating procedures (SOPs), implementation of safe working practices, and project presentation. These activities provided opportunities for students to develop cognitive understanding, professional attitudes, and practical competencies related to occupational safety within realistic agricultural workplace contexts.

### 3.3 Research instruments

Three complementary instruments were employed to evaluate students’ learning outcomes across the cognitive, affective, and psychomotor domains ( Table 2). Each instrument was designed to represent a different dimension of occupational safety competence. The cognitive domain was measured using an achievement test developed according to the learning objectives of occupational safety instruction. The test evaluated students’ conceptual understanding of hazard identification, occupational safety principles, risk assessment, and standard operating procedures. The affective domain was assessed using an observation sheet completed during project implementation. The observation focused on students’ safety attitudes, responsibility, compliance with safety procedures, teamwork, and commitment to maintaining safe working practices throughout the learning activities. The psychomotor domain was evaluated using a performance rubric that assessed students’ practical ability to implement occupational safety procedures during authentic project activities. Performance indicators included the correct use of personal protective equipment, adherence to standard operating procedures, safe operation of equipment, and the ability to identify and control workplace hazards.

**Table 2.** Research instruments.

Variable	Instrument	Main indicator	Assessment technique
Cognitive	Achievement Test	Knowledge of occupational safety principles, hazard identification, risk assessment, and standard operating procedures, ergonomics, hierarchy of controls, OHS culture	Written test
Affective	Observation Sheet	The observation focused on students’ discipline, responsibility, teamwork, occupational safety awareness, and compliance with safety procedures throughout project implementation	Classroom and project observation
Psychomotor	Performance Rubric	The psychomotor domain was evaluated using a performance rubric assessing students’ ability to identify workplace hazards, develop occupational safety procedures (SOPs), produce project outputs integrating occupational safety principles, communicate project results, and correctly use personal protective equipment (PPE).	Performance assessment

Prior to implementation, the instruments were reviewed by experts in occupational safety and vocational education to establish content validity. The cognitive test consisted of 20 multiple-choice items, while the affective observation sheet comprised 4 indicators, and the psychomotor rubric included 5 performance criteria aligned with the OS-PjBL framework. A description of each instrument is presented in Table 2.

### 3.4 Research procedures

The instructional intervention was implemented using the validated Occupational Safety-Integrated Project-Based Learning (OS-PjBL) model during practical agricultural learning activities. The intervention was designed to embed occupational safety principles throughout every stage of project implementation, enabling students to develop conceptual understanding, professional attitudes, and practical competencies simultaneously. Before the intervention, students completed a cognitive pre-test to determine their initial understanding of occupational safety concepts. Throughout the learning process, students participated in collaborative project activities while their affective and psychomotor performance was continuously observed using structured observation sheets and performance rubrics. At the end of the intervention, students completed a cognitive post-test, and the observation data were summarized to evaluate overall achievement across the three learning domains.

The implementation procedure consisted of five sequential stages, beginning with contextual problem identification and ending with reflection and evaluation. At each stage, occupational safety was integrated as an essential component of project activities rather than being introduced as supplementary instructional content. Table 3 summarizes the instructional procedures implemented during the study.

**Table 3.** Implementation procedure of occupational safety-Integrated project-based learning (OS-PjBL).

Stage	Learning activities	Occupational Safety integration	Learning domain facilitated
**Contextual Problem Identification**	Students identified authentic agricultural problems, analyzed project requirements, and discussed project objectives collaboratively.	Students identified potential workplace hazards, analyzed occupational risks, and determined safety objectives before initiating project activities.	Cognitive
**Project Planning**	Students designed project plans, allocated responsibilities, selected resources, and prepared implementation strategies.	Students developed Standard Operating Procedures (SOPs), selected appropriate Personal Protective Equipment (PPE), and prepared risk-control strategies.	Cognitive & Affective
**Project Implementation**	Students carried out project activities, collected data, developed products, and solved practical problems collaboratively.	Students consistently applied PPE, followed SOPs, implemented hazard control measures, and practiced safe operation of agricultural equipment.	Psychomotor
**Monitoring and Evaluation**	Students monitored project progress, evaluated implementation, discussed emerging problems, and revised project plans when necessary.	Students evaluated compliance with safety procedures, identified unsafe practices, and implemented corrective actions to minimize workplace risks.	Affective & Psychomotor
**Presentation, Reflection, and Improvement**	Students presented project outcomes, reflected on their learning experiences, and discussed project improvements.	Students reflected on occupational safety practices, evaluated safety performance throughout the project, and proposed recommendations for future workplace improvements.	Cognitive, Affective & Psychomotor

Throughout the intervention, occupational safety was treated as an organizing principle that guided every stage of project implementation rather than as an isolated instructional topic. Consequently, students were continuously required to integrate hazard identification, risk assessment, safe decision-making, and practical safety procedures while completing authentic agricultural projects. This continuous integration was expected to facilitate balanced development across the cognitive, affective, and psychomotor domains, providing the basis for the comparative analysis presented in the subsequent section.

### 3.5 Data analysis

The collected data were analyzed using descriptive statistical techniques to examine students’ learning outcomes across the cognitive, affective, and psychomotor domains following the implementation of Occupational Safety-Integrated Project-Based Learning (OS-PjBL). The analysis focused on describing the level of achievement in each learning domain and identifying the domain that demonstrated the highest level of performance after the instructional intervention.

Cognitive learning outcomes were analyzed by calculating the mean, standard deviation, minimum score, and maximum score obtained from the pre-test and post-test. These descriptive statistics were used to illustrate changes in students’ conceptual understanding of occupational safety principles following participation in OS-PjBL.

The affective and psychomotor domains were analyzed using percentage scores derived from structured observation sheets and performance rubrics completed during project implementation. Percentage values for each indicator were calculated to describe students’ achievement in safety awareness, responsibility, teamwork, discipline, hazard identification, implementation of standard operating procedures (SOPs), use of personal protective equipment (PPE), and other occupational safety competencies. The resulting percentages were interpreted using predetermined performance categories to indicate the quality of students’ learning outcomes.

To address the research questions, a comparative descriptive analysis was conducted by comparing the overall achievement obtained in the cognitive, affective, and psychomotor domains. Comparative tables were used to identify similarities and differences among the three domains and to determine which domain demonstrated the highest level of achievement following the implementation of Occupational Safety-Integrated Project-Based Learning. This analytical approach provides a comprehensive description of the multidimensional learning outcomes associated with the instructional model without emphasizing statistical significance testing. Given the descriptive objective of the study, inferential statistical testing was not performed. Instead, the analysis focused on describing and comparing achievement levels across the three learning domains.

## 4. Results

The effectiveness of the Occupational Safety-Integrated Project-Based Learning (OS-PjBL) model was evaluated through its implementation involving 30 students enrolled in the Agricultural Production Technology program. The evaluation focused on three complementary learning domains: cognitive, affective, and psychomotor. Overall, the findings demonstrate that the implementation of OS-PjBL resulted in substantial improvements across all domains, indicating that the instructional model effectively supported the development of holistic occupational safety competence.

The improvement was evident not only in students’ conceptual understanding of occupational safety principles but also in the development of positive safety attitudes and practical competencies required during authentic project implementation. The combination of achievement tests, classroom observations, and performance assessments provides complementary evidence that OS-PjBL promoted balanced learning outcomes aligned with the objectives of vocational education.

### 4.1 Improvement in cognitive learning outcomes

To examine changes in conceptual understanding, students’ cognitive performance was summarized using descriptive statistics before and after the implementation of OS-PjBL. Table 4 presents the distribution of students’ cognitive scores.

**Table 4.** Descriptive statistics of cognitive learning outcomes.

Variable	Pre-test	Post-test
N	30	30
Mean	**52.67**	**86.7**
Standard Deviation	6.62	4.83
Minimum	40	78
Maximum	65	95

The descriptive statistics indicate a marked improvement in students’ conceptual understanding following the implementation of OS-PjBL. Besides the substantial increase in the mean score, the reduction in score dispersion suggests that students achieved more consistent levels of cognitive performance after participating in authentic project activities. This pattern indicates that the instructional model benefited not only high-performing students but also those with initially lower levels of occupational safety knowledge.

### 4.2 Development of affective and psychomotor competencies

Although all affective indicators reached the “Very Good” category ( Table 5), Safety Awareness demonstrated the highest achievement (98%), indicating that continuous exposure to authentic workplace situations successfully strengthened students’ sensitivity toward occupational safety. Meanwhile, Discipline showed the lowest percentage (90%), suggesting that maintaining consistent procedural compliance remains relatively more challenging than developing general safety awareness. Tables 5 and 6 summarize students' affective and psychomotor achievements observed throughout the implementation of OS-PjBL.

**Table 5.** Affective learning outcomes.

Indicator	Percentage	Category
Discipline	90%	Very Good
Responsibility	93%	Very Good
Teamwork	93%	Very Good
Safety Awareness	**98%**	Very Good
**Overall**	**93%**	Very Good

Students achieved consistently high performance across all practical indicators ( Table 6). The highest score obtained for PPE usage (98%) indicates that repeated engagement with authentic project activities successfully reinforced safe working practices. Similarly, the consistently high achievement across hazard identification, SOP development, product quality, and presentation suggests that students were able to integrate occupational safety principles into practical project implementation.

**Table 6.** Psychomotor learning outcomes.

Indicator	Percentage	Category
Hazard Identification	93%	Very Skilled
SOP Development	93%	Very Skilled
Product Quality	95%	Very Skilled
Presentation	95%	Very Skilled
PPE Usage	**98%**	Very Skilled

### 4.3 Comparative analysis across learning domains

**Figure 2.**
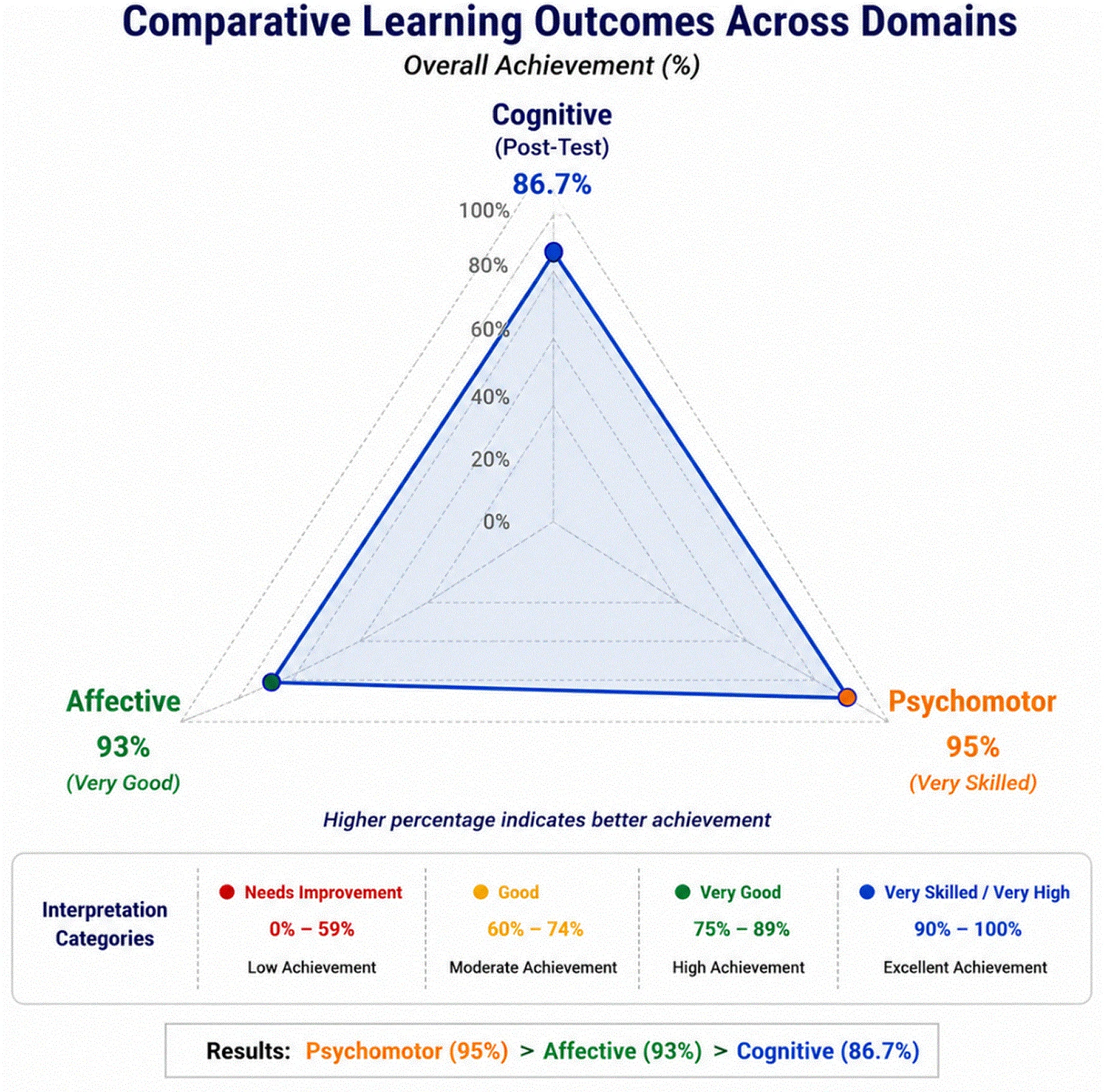
Comparative learning outcomes across domains.

To address the primary objective of this study, learning outcomes were compared across the cognitive, affective, and psychomotor domains. The comparison indicates that OS-PjBL positively influenced all domains; however, the magnitude of improvement differed among them ( Figure 2). Cognitive learning demonstrated a high level of conceptual improvement (Post Test = 86.7), while affective competence reached an overall achievement of 93%. The strongest outcome was observed in the psychomotor domain, where students achieved an overall performance score of 95%.

The comparison reveals a clear pattern across the three learning domains. While students achieved high performance in every domain, psychomotor competence demonstrated the highest level of achievement, followed by affective competence and cognitive understanding. This pattern suggests that authentic project activities were particularly effective in facilitating competencies directly associated with practical workplace performance.

Despite the differences in achievement levels, the consistently high performance observed across all domains indicates that Occupational Safety-Integrated Project-Based Learning supported balanced competence development. The findings therefore suggest that integrating occupational safety into authentic project experiences contributes not only to conceptual learning but also to the development of professional attitudes and practical workplace skills.

## 5. Discussion

### 5.1 Occupational safety-Integrated project-based learning enhances holistic learning outcomes

The findings of this study demonstrate that Occupational Safety-Integrated Project-Based Learning (OS-PjBL) effectively promotes holistic learning outcomes among agricultural vocational students. Improvements were consistently observed across the cognitive, affective, and psychomotor domains, indicating that integrating occupational safety into project-based activities supports comprehensive competency development rather than isolated knowledge acquisition. The learning process enabled students not only to understand occupational safety concepts but also to develop professional attitudes and demonstrate practical competencies through authentic workplace-related projects. This finding suggests that OS-PjBL provides a learning environment in which knowledge, attitudes, and skills are developed simultaneously through meaningful learning experiences.

These findings can be interpreted through the perspective of experiential learning theory, which emphasizes that knowledge is constructed through direct experience and reflective practice. Rather than receiving occupational safety concepts through conventional lectures, students actively identified workplace hazards, analyzed risks, developed safety procedures, and evaluated their project outcomes. Such activities encouraged learners to transform theoretical knowledge into practical understanding while simultaneously strengthening professional attitudes toward workplace safety. Consequently, learning became more contextual, meaningful, and closely aligned with the characteristics of vocational education.

The present findings are consistent with previous studies reporting that Project-Based Learning enhances students’ conceptual understanding ( Girgin & Coştu, 2024), collaboration ( Martawijaya, Rahmadhanningsih, et al., 2023b; Naila Rohmaniyah & Siti Wulan Asih, 2024), and practical skills through authentic learning experiences ( Wang et al., 2025). However, the current study extends previous evidence by embedding occupational safety principles throughout every stage of project implementation. In many previous PjBL studies, safety is treated merely as procedural guidance during practical activities. In contrast, the present model positions occupational safety as a central learning objective, requiring students to continuously integrate hazard identification, risk assessment, and safety decision-making into project completion. This integration allows students to develop occupational safety competence as an inseparable component of vocational expertise rather than as an additional instructional topic.

From the perspective of vocational education, these findings reinforce the importance of integrating authentic workplace contexts into instructional design. Agricultural vocational education requires graduates who are not only technically competent but also capable of maintaining safe working practices in environments characterized by relatively high occupational risks. Therefore, integrating occupational safety within project-based learning provides a more comprehensive approach for preparing students to meet professional expectations while fostering holistic competence that encompasses knowledge, attitudes, and practical performance.

### 5.2 Why did psychomotor competence improve the most?

Among the three learning domains, psychomotor competence demonstrated the highest level of achievement following the implementation of OS-PjBL. This finding indicates that authentic project-based activities provide particularly strong opportunities for students to transform conceptual understanding into practical workplace performance. Through continuous engagement in hazard identification, safety inspections, development of standard operating procedures (SOPs), appropriate use of personal protective equipment (PPE), and project presentations, students repeatedly practiced occupational safety procedures within realistic learning situations. These experiences likely contributed to the superior performance observed in the psychomotor domain.

The predominance of psychomotor improvement can be explained through the principle of *learning by doing*, which underpins vocational education. Practical competence develops most effectively when learners repeatedly perform authentic tasks that closely resemble professional practice. Within the OS-PjBL model, students were not passive recipients of safety information but active participants responsible for identifying workplace risks, implementing preventive measures, and producing safety-related products. Such repeated practice enables procedural knowledge to become increasingly automatic, thereby strengthening technical competence alongside confidence in applying occupational safety procedures.

These findings are consistent with previous research in vocational and technical education showing that project-based and performance-based learning environments are particularly effective in improving practical competencies ( Chrismondari et al., 2025; Evains et al., 2024). Authentic learning experiences encourage students to integrate theoretical understanding with hands-on application, leading to stronger performance than instructional approaches that rely primarily on lectures or demonstrations ( Newmann et al., 1996; Palloan & Swandi, 2019). The present findings further suggest that occupational safety education benefits substantially from this experiential approach because safe work behaviour can only be fully developed through repeated practice within realistic contexts rather than through theoretical instruction alone.

An important contribution of this study is the demonstration that occupational safety can serve not only as instructional content but also as a framework for developing professional competence. By embedding safety principles throughout the entire project cycle, students learned to perform technical tasks while simultaneously considering workplace risks and appropriate preventive actions. This integrated approach reflects the expectations of modern agricultural industries, where technical expertise and safety performance are inseparable components of professional competence. Consequently, the findings highlight the value of designing vocational learning environments that combine authentic project experiences with systematic occupational safety integration to prepare graduates for increasingly complex workplace demands.

### 5.3 Safety culture as an educational outcome

Beyond improving students’ cognitive understanding and practical competencies, the findings indicate that Occupational Safety-Integrated Project-Based Learning (OS-PjBL) contributes to the development of a positive safety culture among vocational students. High levels of discipline, responsibility, teamwork, and safety awareness suggest that occupational safety was not merely learned as theoretical knowledge but gradually internalized as part of students’ professional behaviour during project implementation. This outcome is particularly important because effective occupational safety education aims not only to increase knowledge but also to cultivate attitudes and behaviours that support safe working practices.

The development of safety culture can be explained by the continuous integration of occupational safety principles throughout the learning process. During project activities, students were required to identify workplace hazards, comply with safety procedures, use appropriate personal protective equipment, and collaboratively evaluate potential risks before completing project tasks. These repeated experiences encouraged students to perceive safety not as an external requirement imposed by instructors but as an integral component of professional responsibility. Such learning experiences are consistent with the view that safety culture develops gradually through repeated practice, shared responsibility, and reflective learning rather than through isolated classroom instruction.

previous studies have emphasized that safety education in vocational institutions often focuses primarily on procedural compliance and regulatory knowledge. While such approaches are essential, they may be insufficient for developing long-term safety behaviours if students rarely experience authentic workplace situations. The present findings suggest that embedding occupational safety within project-based learning provides opportunities for students to practice safe decision-making repeatedly while working collaboratively to solve authentic problems. Consequently, occupational safety becomes embedded within students’ everyday learning activities instead of remaining a separate instructional topic.

An important contribution of this study is the demonstration that safety culture can be developed simultaneously with academic learning through authentic project experiences. Rather than separating technical competence from workplace safety, the OS-PjBL model integrates both dimensions into a unified instructional framework. This integration supports the preparation of vocational graduates who are not only technically competent but also capable of demonstrating responsible and safety-oriented professional behaviour within agricultural workplaces.

### 5.4 Implications for agricultural vocational education

The findings of this study have several implications for vocational education, particularly for institutions preparing graduates to work in high-risk agricultural environments. First, occupational safety should be integrated throughout vocational learning activities rather than being taught as an isolated theoretical course. Embedding safety principles within project implementation enables students to continuously connect conceptual knowledge with authentic workplace practices, thereby strengthening both technical competence and professional responsibility.

Second, the findings suggest that assessment in vocational education should extend beyond conventional written examinations. While cognitive achievement remains important, comprehensive evaluation should also include students’ attitudes toward occupational safety and their practical performance during authentic workplace tasks. Such multidimensional assessment provides a more comprehensive representation of vocational competence and better reflects the expectations of modern industries, where technical performance and workplace safety are inseparable.

Third, the present study highlights the importance of authentic project-based learning for strengthening graduate employability. Agricultural industries increasingly require workers who can identify workplace hazards, manage occupational risks, collaborate effectively within multidisciplinary teams, and consistently implement safe working procedures. Therefore, vocational curricula should provide learning experiences that closely resemble professional practice, allowing students to develop these competencies before entering the workforce.

Finally, the OS-PjBL model may also serve as a practical instructional framework for vocational educators seeking to strengthen occupational safety education without increasing curriculum load. Instead of introducing additional safety courses, instructors can integrate occupational safety principles into existing project activities, making safety education more contextual, meaningful, and sustainable across vocational programs.

### 5.5 Limitations and future research

Several limitations should be considered when interpreting the findings of this study. First, the implementation of the OS-PjBL model was conducted within a single agricultural vocational institution involving students from one study program. Although the findings provide encouraging evidence regarding the effectiveness of the model, the generalizability of the results to other vocational disciplines or educational contexts remains limited.

Second, this study primarily evaluated short-term learning outcomes immediately following the implementation of the instructional model. Consequently, the extent to which improvements in cognitive achievement, safety attitudes, and practical competencies are maintained over time has not yet been examined. Longitudinal studies are therefore needed to investigate the sustainability of occupational safety competencies after students complete vocational training or transition into professional employment.

Third, the study employed a one-group pretest-posttest approach to evaluate learning effectiveness. Although significant improvements were observed across all learning domains, future research employing quasi-experimental or randomized controlled designs would provide stronger causal evidence regarding the effectiveness of Occupational Safety-Integrated Project-Based Learning compared with conventional instructional approaches.

Future research may also extend the present work by implementing the OS-PjBL model across different vocational disciplines, including engineering, manufacturing, health sciences, and construction education, where occupational safety represents a fundamental professional competency. Comparative studies across vocational sectors would provide valuable evidence regarding the adaptability and effectiveness of the model in diverse educational settings.

In addition, future investigations could integrate emerging digital technologies such as virtual reality (VR), augmented reality (AR), artificial intelligence (AI), and learning analytics to support occupational safety training. These technologies have the potential to provide immersive simulations of hazardous workplace situations, enabling students to develop safety competencies within controlled and realistic learning environments. Such developments would further strengthen the contribution of OS-PjBL toward preparing vocational graduates capable of meeting the increasingly complex occupational safety demands of Industry 5.0.

## 6. Conclusion

This study investigated the effectiveness of Occupational Safety-Integrated Project-Based Learning (OS-PjBL) in improving cognitive, affective, and psychomotor learning outcomes among students in agricultural vocational education. The findings demonstrate that integrating occupational safety principles into project-based learning effectively promotes holistic competency development. Students showed substantial improvements in conceptual understanding of occupational safety, developed positive professional attitudes characterized by discipline, responsibility, teamwork, and safety awareness, and demonstrated high levels of practical competence during authentic project implementation. These findings indicate that occupational safety can be effectively embedded within project-based learning to support balanced learning across multiple educational domains rather than focusing solely on theoretical knowledge acquisition.

The study further reveals that the greatest learning improvement occurred in the psychomotor domain, suggesting that authentic project experiences provide particularly strong opportunities for students to translate occupational safety knowledge into workplace performance. Simultaneously, the high achievement observed in the affective domain indicates that continuous engagement with authentic occupational safety practices contributes to the development of a positive safety culture among vocational students. These findings highlight that occupational safety education should not be viewed as a separate instructional component but rather as an integral element of vocational learning that simultaneously strengthens technical competence, professional behaviour, and workplace readiness.

This study contributes to the growing literature on project-based learning by proposing and empirically validating an instructional approach in which occupational safety functions as the organizing principle of authentic learning activities rather than as supplementary instructional content. The proposed OS-PjBL model extends conventional project-based learning by integrating hazard identification, risk assessment, safety decision-making, and performance-based assessment throughout the entire learning process. Consequently, the model offers a practical pedagogical framework for vocational institutions seeking to prepare graduates who are not only technically competent but also capable of working safely and responsibly in increasingly complex agricultural work environments. These findings provide empirical support for embedding occupational safety systematically within vocational curricula as a strategy for developing competent, safety-conscious, and industry-ready professionals.

## Ethical consideration

The authors stated that the study was approved by the Institutional Review Board at Universitas Bosowa on 3 July 2025 with the reference number 173b/1.02/DRIPM-UNIBOS/VII/2025. Written informed consents were obtained from the participants.

## AI statement

The authors stated that generative artificial intelligence tools were used in a limited manner to assist with language editing, organization of academic writing, improvement of clarity, and improving image quality. The conceptual framework, research design, data analysis, interpretation, and final conclusions were developed entirely by the authors.

## Data Availability

The data supporting the findings of this study are available in figshare:
• Cognitive Pre-Test & Post-Test Instrument. DOI:
https://doi.org/10.6084/m9.figshare.33090311 ( Basma, 2026b)
• Affective Observation Sheet. DOI:
https://doi.org/10.6084/m9.figshare.33090368 ( Basma, 2026a)
• Project Performance and Product Assessment Rubric (Psychomotor). DOI:
https://doi.org/10.6084/m9.figshare.33090392 ( Basma, 2026c). Data are available under the terms of the Creative Commons Attribution 4.0 International (CC-BY 4.0) license.
