# Supplementary material for: Which Learning Domains Benefit Most from Occupational Safety-Integrated Project-Based Learning? A Comparative Analysis of Cognitive, Affective, and Psychomotor Outcomes in Agricultural Vocational Education
Source: F1000Res. 2026 Sep 2;15:1305. Originally published 2026 Aug 6. [Version 2] doi: 10.12688/f1000research.187948.2 (PMC13562939; doi:10.12688/f1000research.187948.2)
Supplement: Peer review history

# Reviewer report: Reviewer response for version 2

**DOI:** 10.5256/f1000research.209199.r517302

**Published:** 2026-09-04

**Authors:** Marisol Jane M Beray

**Affiliations:** 1 Caraga State University, Cabadbaran City, Agusan del Norte, Philippines

The authors have thoroughly revised the paper in response to the comments and suggestions previously provided, successfully addressing the concerns raised.

Is the work clearly and accurately presented and does it cite the current literature?

Yes

If applicable, is the statistical analysis and its interpretation appropriate?

Partly

Are all the source data underlying the results available to ensure full reproducibility?

Yes

Is the study design appropriate and is the work technically sound?

Yes

Are the conclusions drawn adequately supported by the results?

Yes

Are sufficient details of methods and analysis provided to allow replication by others?

Yes

Reviewer Expertise:

technology education, electronics engineering technology, pedagogy, generative artificial intelligence

I confirm that I have read this submission and believe that I have an appropriate level of expertise to confirm that it is of an acceptable scientific standard.

# Reviewer report: Reviewer response for version 1

**DOI:** 10.5256/f1000research.207427.r509355

**Published:** 2026-08-22

**Authors:** Marisol Jane M Beray

**Affiliations:** 1 Caraga State University, Cabadbaran City, Agusan del Norte, Philippines

To strengthen the statistical analysis and interpretation, please include a separate table that clearly outlines the scoring and quantification of data. This addition will make the methodological basis of the results in Section 4 more transparent and easier for readers to follow. Also, the manuscript would benefit from a deeper synthesis of prior emperical findings in the literature review and an expanded discussion of implications for broader TVET contexts. 

Is the work clearly and accurately presented and does it cite the current literature?

Yes

If applicable, is the statistical analysis and its interpretation appropriate?

Partly

Are all the source data underlying the results available to ensure full reproducibility?

Yes

Is the study design appropriate and is the work technically sound?

Yes

Are the conclusions drawn adequately supported by the results?

Yes

Are sufficient details of methods and analysis provided to allow replication by others?

Yes

Reviewer Expertise:

technology education, electronics engineering technology, pedagogy, generative artificial intelligence

I confirm that I have read this submission and believe that I have an appropriate level of expertise to confirm that it is of an acceptable scientific standard, however I have significant reservations, as outlined above.

# Response

**Published:** 2026-08-23

**Authors:** Basma Basma

**Affiliations:** Postgraduate Program, State University of Makassar, Makassar, South Sulawesi, Indonesia

Thank you very much for this valuable and constructive suggestion. We have carefully revised the manuscript to improve the transparency of the scoring and data quantification procedures, strengthen the synthesis of previous empirical findings, and broaden the discussion of the implications for TVET contexts.

 First, we added a separate table entitled **“Table 3. Scoring and Quantification of Learning Outcomes”** in the Methods section. The table explicitly presents the assessment instrument, assessment components, scoring procedures, maximum scores, and quantification formulas for the cognitive, affective, and psychomotor domains. The cognitive domain is based on a 20-item multiple-choice test, while the affective and psychomotor domains are quantified using their respective observation and performance assessment criteria. We also added a detailed explanation following the table to clarify how the raw scores were converted into a common 0–100 scale. This revision makes the methodological basis of the quantitative results presented in Section 4 more transparent and facilitates comparison across the three learning domains.

 Second, we substantially revised the literature review by adding a new section entitled **“2.4 Empirical Synthesis of Project-Based Learning and Occupational Safety Education.”** This section goes beyond listing previous studies by synthesizing empirical findings concerning the effectiveness of Project-Based Learning in vocational and technical education, evidence concerning occupational safety education, and the relationship between authentic project experiences and the development of vocational competencies. The revised discussion also identifies the limited integration of Project-Based Learning and occupational safety education and clarifies how the present study addresses this gap by examining cognitive, affective, and psychomotor outcomes simultaneously.

 Third, we expanded the discussion of implications in **Section 5.4, “Implications for TVET Practice.”** The revised section retains the agricultural vocational context of the present study while discussing the potential relevance of the OS-PjBL approach to broader TVET sectors. In particular, we discuss how the integration of occupational safety into authentic project-based learning and multidimensional assessment may be adapted to other occupational contexts, including manufacturing, construction, automotive, engineering, and other technical fields. We also emphasize that the specific projects, safety competencies, and assessment criteria should be adapted to the characteristics and risk profiles of each TVET sector.

 In addition, we refined the interpretation of the comparative results to distinguish **cognitive improvement** from **affective and psychomotor achievement levels**, given that pre-test and post-test measurements were available for the cognitive domain, whereas the affective and psychomotor domains were assessed through observation and performance assessment. Accordingly, cross-domain comparisons are presented as descriptive comparisons of achievement levels rather than as statistically tested differences in improvement.

 We sincerely appreciate the reviewer’s suggestion, which has helped us improve the methodological transparency, empirical grounding, and broader relevance of the manuscript.

# Reviewer report: Reviewer response for version 1

**DOI:** 10.5256/f1000research.207427.r509359

**Published:** 2026-08-21

**Authors:** Nurhayati Nurhayati

**Affiliations:** 1 Singkawang Teacher Training and Education College (STKIP), Instiut Sains dan Bisnis Internasional Singkawang, Singkawang, West Kalimantan, Indonesia

The research article is very good and requires only minor revisions. The statistics presented are descriptive statistics because the research used is development. The sample used could be larger, because the 30 samples used for development research cannot yet describe the results that have an impact. The conclusions given are based on the research results presented.

Is the work clearly and accurately presented and does it cite the current literature?

Yes

If applicable, is the statistical analysis and its interpretation appropriate?

Not applicable

Are all the source data underlying the results available to ensure full reproducibility?

Yes

Is the study design appropriate and is the work technically sound?

Yes

Are the conclusions drawn adequately supported by the results?

Yes

Are sufficient details of methods and analysis provided to allow replication by others?

Yes

Reviewer Expertise:

Mathematic Education

I confirm that I have read this submission and believe that I have an appropriate level of expertise to confirm that it is of an acceptable scientific standard.
